# 

**Published:** 1894-04

**Authors:** 


### TEXAS STATE MEDICAL ASSOCIATION.

The following circular has been issued:

AUSTIN, TEXAS, March 10, 1894.

The undersigned, Chairman of Committee on Hotels, takes pleasure in announcing to fellow-members of the Texas State Medical Association, who may attend the next annual meeting of the Association at Austin, Texas, April 26, 1894, that he has secured for them commuted rates at the principal hotels of Austin, as follows:

Driskill, board and lodging per diem....................$2 50
Hotel Salge, board and lodging per diem................ 2 00
Avenue Hotel, board and lodging per diem.............. 2 00
Hotel Orr, board and lodging per diem.................. 1 50
Austin House, board and lodging per diem.............. 1 25

The condition of the foregoing commuted rates is, that physicians attending the T. S. M. Association must lodge at least two in each room.

Members desiring to secure rooms in advance, in hotel or private boarding houses will please drop us a line stating their wishes, and we will take great pleasure in promptly complying with their requests.

Our city is generously provided with good hotels and nice boarding houses, so we can easily accommodate, in comfortable and elegant style, the large number of members we hope will meet with us.

Come, brethern, and let us have a royal good time. We certainly earn one holiday in a year; come and help us enjoy it, with a "feast of reason and a flow of soul."

Yours most cordially,

Q. C. SMITH, M. D.,
Chairman of Committee on Hotels.

617 Colorado Street.

